# Recurrent Ischemic Strokes in a Young Female: An Atypical Initial Presentation of Systemic Lupus Erythematosus

**DOI:** 10.7759/cureus.93245

**Published:** 2025-09-26

**Authors:** Felicita M Tayong, Tanzeela Shah, Ayaz Ali, Phillip George, Nida Gul, Aysha Habib, Maria Faryal, Guillermo Andrés Moreno Cortes

**Affiliations:** 1 Neurosurgery, University of Florida College of Medicine, Gainesville, USA; 2 College of Medicine, University of Science, Arts and Technology College of Medicine, British West Indies, MSR; 3 Medicine, Lady Reading Hospital, Peshawar, PAK; 4 Internal Medicine, Khyber Medical College, Peshawar, PAK; 5 Family Medicine, Ross University School of Medicine, Bridgetown, BRB; 6 Internal Medicine, Saidu Medical College, Swat, PAK; 7 Medicine, Khyber Medical University Institute of Medical Sciences, Kohat, PAK; 8 Family Medicine, Universidad del Rosario, School of Medicine and Health Sciences, Bogota, COL

**Keywords:** autoimmune disease, neuropsychiatric sle, recurrent stroke, systemic lupus erythematosus, young female

## Abstract

Systemic lupus erythematosus (SLE) is a chronic autoimmune disease that can affect multiple organs, but recurrent ischemic strokes as the first presentation are very rare. We report the case of a 35-year-old woman who presented with sudden loss of consciousness and right-sided weakness, with a history of multiple previous strokes of unknown cause. Brain magnetic resonance imaging (MRI) revealed multifocal subacute ischemia with hemorrhagic transformation, while laboratory findings showed thrombocytopenia, anemia, raised erythrocyte sedimentation rate (ESR), impaired renal function, and a positive antinuclear antibody (ANA) test, confirming the diagnosis of SLE with neuropsychiatric involvement. She was treated with intravenous steroids, antibiotics, and supportive care, with gradual clinical improvement. This case highlights the importance of considering autoimmune diseases like SLE in young patients with unexplained recurrent strokes, as early diagnosis and immunosuppressive therapy can prevent further complications and improve outcomes.

## Introduction

Relapsing-remitting symptoms, autoantibodies, and systemic inflammation are the hallmarks of systemic lupus erythematosus (SLE). SLE is characterised by multi-system involvement and primarily affects young women. SLE frequently targets the central nervous system (CNS), and when it does, the morbidity and mortality rates rise [1].

Rapidly developed clinical signs of focal or global disturbance of cerebral function, lasting more than 24 hours or leading to death, with no apparent cause other than of vascular origin, is how the World Health Organisation defines stroke. Stroke is regarded as the second greatest cause of mortality globally and is one of the main causes of disability. Between 1990 and 2019, the incidence of stroke rose by 70%, while the stroke fatality rate increased to 43% [2].

Stroke episodes are common among SLE patients, affecting 3-20% of them. They typically occur within the first five years after diagnosis and primarily impact young people [3].

Patients with SLE are eight times more likely to have a stroke than the general population, and their risk of cardiovascular events rises by 3% annually after diagnosis. Twenty to 30 percent of SLE patients die as a result of these factors [4].

While SLE is a multisystem autoimmune disease known to increase the risk of cerebrovascular events, ischemic stroke is more commonly seen as a late complication rather than an initial manifestation. Neurological involvement, such as stroke, typically occurs after the diagnosis of SLE has been established. However, recurrent ischemic strokes as the first clinical presentation of SLE remain extremely rare.

This case highlights the importance of considering underlying autoimmune disorders, particularly SLE, in young patients presenting with unexplained recurrent strokes, especially in the absence of traditional vascular risk factors. The incidental discovery of SLE in our patient following recurrent cerebrovascular events underscores the need for high clinical suspicion and comprehensive evaluation in atypical stroke cases. Early recognition is crucial to initiate appropriate immunosuppressive therapy and prevent further complications.

## Case presentation

A 35-year-old female presented to the medical emergency department with a history of sudden loss of consciousness and right-sided weakness involving both the upper and lower limbs. The symptoms began abruptly, and there was no reported preceding trauma, seizure activity, or chest pain. She was brought to the hospital by her family in an obtunded state. On presentation, her Glasgow Coma Scale (GCS) was 9/15, and she exhibited flaccid weakness on the right side, consistent with a possible cerebrovascular event.

Initial vital signs were within acceptable ranges: blood pressure 110/70 mmHg, respiratory rate 26 breaths per minute, oxygen saturation 94% on nasal cannula, and random blood sugar (RBS) 145 mg/dL. There was no clinical evidence of hypoglycemia or hypertensive crisis. The patient was promptly stabilized with oxygen support, intravenous fluids, and monitoring, while preparations for imaging and laboratory workup were initiated.

She had a known history of recurrent cerebrovascular accidents (CVAs), with three documented ischemic stroke episodes over the past several years. Despite multiple hospital visits, no clear etiology had been established, and she had not been maintained on long-term antiplatelet or anticoagulant therapy. There was no history of smoking, diabetes mellitus, or overt cardiac illness.

On neurological examination, she had right-sided hemiparesis with reduced tone and an absent plantar reflex on the right. Her pupils were equal and reactive, and there were no signs of meningeal irritation. She was drowsy but arousable, with intermittent episodes of confusion. Cardiovascular and abdominal examinations were unremarkable. However, chest auscultation revealed basal crackles, and during admission, she developed features suggestive of aspiration pneumonia, likely related to impaired swallowing due to her neurological deficits.

Laboratory investigations revealed multiple abnormalities. Complete blood count (CBC) showed thrombocytopenia and normocytic anemia. Renal function tests (RFTs) were deranged, and the erythrocyte sedimentation rate (ESR) was markedly elevated. Most notably, her antinuclear antibody (ANA) test was positive, prompting further autoimmune evaluation. These findings were highly suggestive of an underlying autoimmune condition, most likely SLE (refer to Table 1 for detailed laboratory findings).

**Table 1:** Laboratory parameters showing autoimmune activity, hematological abnormalities, and renal dysfunction RBC: red blood cell, PLT: platelets, HGB: hemoglobin, ESR: erythrocyte sedimentation rate

Parameters	Patient value	Normal range	Interpretation
ANA (antinuclear antibody)	2+ Speckled (1:160 t0 1:400)	Less than 1:40	Moderate positive ANA
Complete blood count			
RBC	2x10.e 6/ul	4-6x10.e 6/ul	Low RBC level
HGB	6.1 g/dl	11.5-17.5 g/dl	Very low HGB (anemic)
PLT	15x10.e 3/ul	150-450x10.e3/ul	Very low platelet count(thrombocytopenia)
Inflammatory marker			
ESR	67 mm/1^st^ hour	0-20 mm/1^st^ hour	Raised
CRP (C-reactive protein)	Less than 0.4 mg/dl	Less than 1 mg/dl	Normal
Renal function tests			
Blood urea	136.64 mg/dl	18-45 mg/dl	Deranged
Creatinine	1.08 mg/dl	0.42-1.06 mg/dl	Deranged

**Figure 1:**
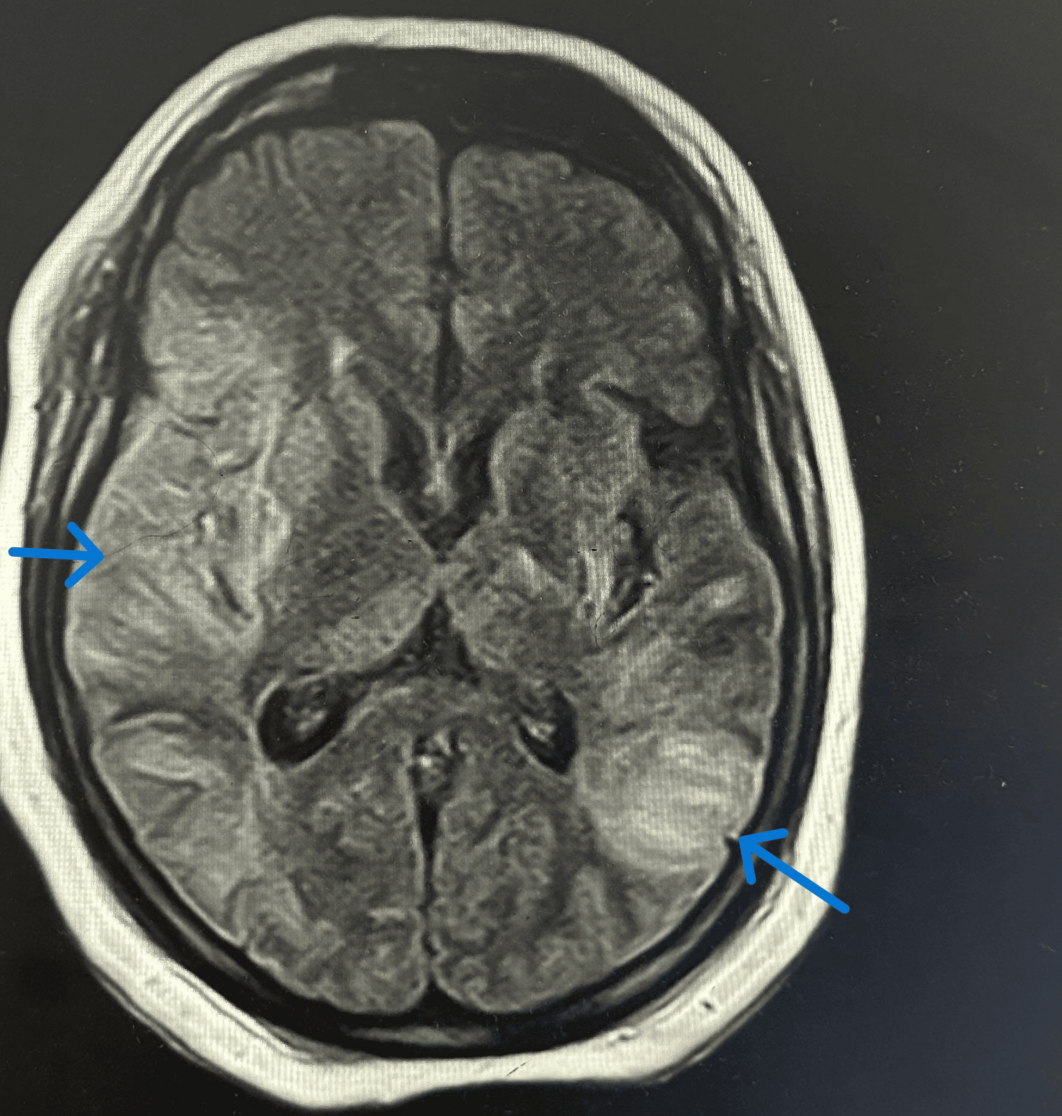
MRI of the brain showing ischemic changes in both cerebral hemispheres.

**Figure 2:**
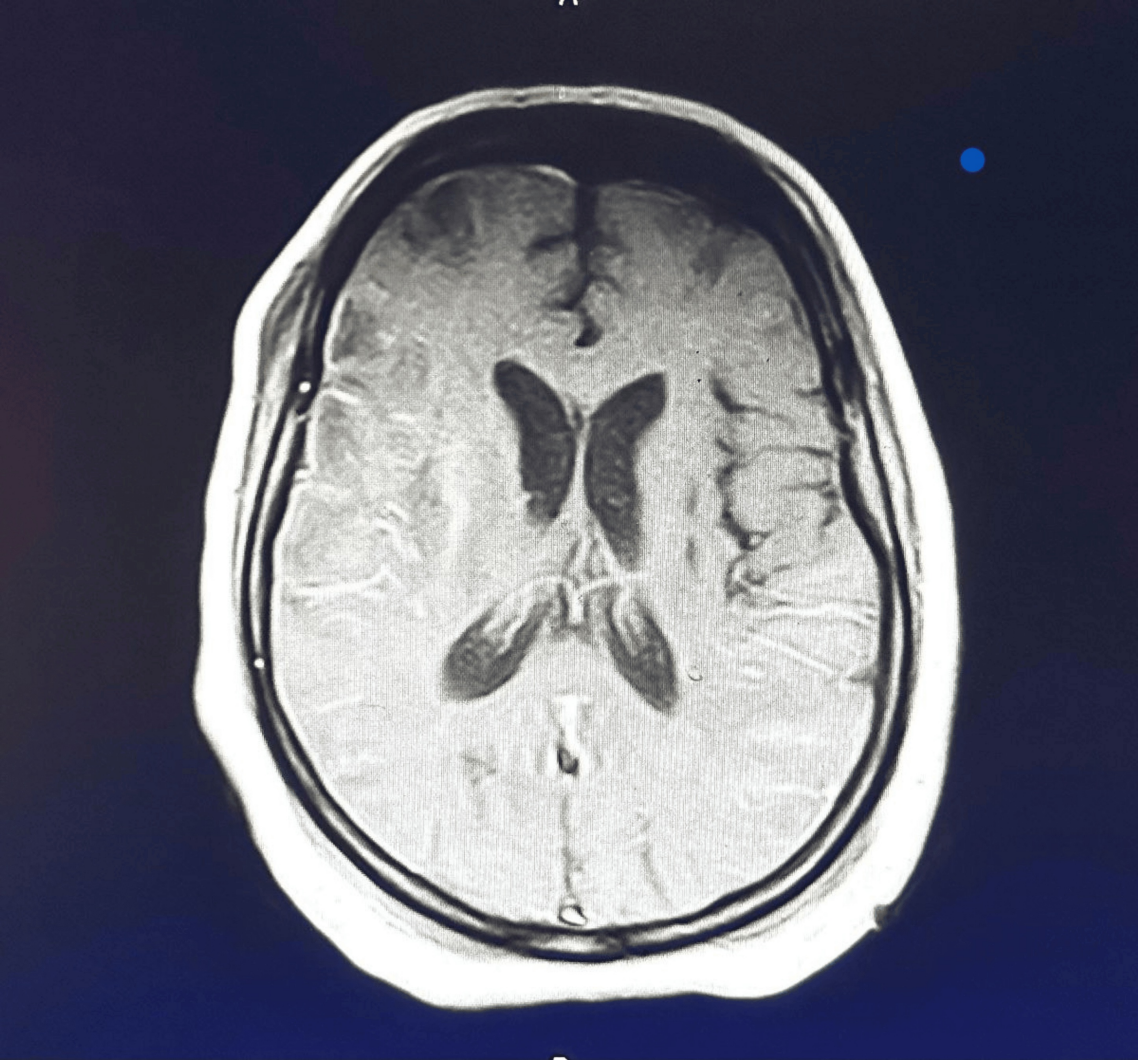
MRI of the brain shows ischemic changes.

A magnetic resonance imaging (MRI) scan of the brain revealed multiple regions of restricted diffusion in the right frontal, parietal, temporal, and occipital lobes, as well as the right caudate nucleus and lentiform nucleus. The MRI findings were consistent with subacute multifocal ischemia with evidence of hemorrhagic transformation and lamellar necrosis, indicating both recent and evolving infarcts (see Figures 1-2 for imaging results).

Further testing, including HbA1c, viral serologies, serum electrolytes, ferritin, and thyroid function tests, was within normal limits. There was no evidence of diabetes, infection, or metabolic derangement contributing to her presentation.

The constellation of recurrent strokes, laboratory abnormalities, characteristic MRI findings, and the newly identified positive ANA led to a final diagnosis of SLE with neuropsychiatric involvement.

She was admitted to the medical high-dependency unit and started on intravenous methylprednisolone pulse therapy to address autoimmune inflammation. Metronidazole and clarithromycin were initiated for the management of aspiration pneumonia, and levetiracetam was prescribed for seizure prophylaxis. Supportive treatment with paracetamol was also given for fever and pain control. Her clinical condition began to stabilize over the following days, with gradual neurological improvement and resolution of infectious signs.

## Discussion

This case is particularly notable due to the patient’s history of multiple ischemic strokes occurring prior to any formal diagnosis of SLE. Despite repeated hospitalizations, she was never evaluated for an underlying autoimmune disorder. The absence of conventional vascular risk factors such as hypertension, diabetes, or smoking history further obscured the diagnosis. It was only after the current episode, with a thorough laboratory and imaging workup, that the autoimmune nature of her illness was uncovered. This delay in diagnosis highlights the clinical challenge and underscores the need for broader differential considerations, including autoimmune etiologies, in cases of recurrent stroke in young patients.

An autoimmune laboratory profile is used to support the clinical diagnosis of SLE, a complex multisystem autoimmune illness. The American College of Rheumatology (ACR) established updated criteria in 1997 to aid in the diagnosis of SLE [5]. In 1924, Libman and Sacks introduced nonrheumatic verrucous endocarditis to the SLE symptom complex and reported valve abnormalities in four lupus patients [6]. Libman-Sacks endocarditis consists of sterile fibrofibrinous vegetations, typically developing on the ventricular surface of the mitral valve and most often affecting left-sided heart valves [7]. The disease progresses from varying degrees of inflammation and acute fibrin deposits to end-stage or healed forms with fibrous plaque formation. The pathophysiology is believed to involve the production of fibrin-platelet thrombi, which organize, undergo fibrosis and scarring, and ultimately lead to valve dysfunction. In addition, emboli may reach the brain and result in ischemic stroke [8].

According to several studies, individuals with SLE have a significantly higher risk of both ischemic and hemorrhagic stroke compared with the general population [9]. In SLE, antibodies against endothelial cells are formed, and immune complexes are deposited within vessel walls [10]. These autoantibodies can cause endothelial activation, leading to vasculitis and increased stroke risk. Younger patients demonstrate the highest relative risk of stroke, which may be partially explained by the accelerated and premature atherosclerosis associated with SLE [11].

## Conclusions

This case demonstrates an uncommon and often overlooked initial presentation of SLE-recurrent ischemic strokes in a young female without traditional risk factors. It serves as a critical reminder that autoimmune diseases, particularly SLE, should be strongly considered in the differential diagnosis when encountering young patients with unexplained or recurrent cerebrovascular events. Early recognition and treatment not only mitigate neurological damage but also improve long-term prognosis and quality of life. A multidisciplinary approach involving neurologists, rheumatologists, and internists is essential for optimal patient care in such complex cases.

## References

[1] de Amorim LC, Maia FM, Rodrigues CE (2017). Stroke in systemic lupus erythematosus and antiphospholipid syndrome: risk factors, clinical manifestations, neuroimaging, and treatment. Lupus.

[2] (2021). Global, regional, and national burden of stroke and its risk factors, 1990-2019: a systematic analysis for the Global Burden of Disease Study 2019. Lancet Neurol.

[3] Futrell N, Millikan C (1989). Frequency, etiology, and prevention of stroke in patients with systemic lupus erythematosus. Stroke.

[4] Krishnan E (2005). Stroke subtypes among young patients with systemic lupus erythematosus. Am J Med.

[5] Hochberg MC (1997). Updating the American College of Rheumatology revised criteria for the classification of systemic lupus erythematosus. Arthritis Rheum.

[6] Libman E, Sacks B (1924). A hitherto undescribed form of valvular and mural endocarditis. Arch Intern Med.

[7] Hojnik M, George J, Ziporen L, Shoenfeld Y (1996). Heart valve involvement (Libman-Sacks endocarditis) in the antiphospholipid syndrome. Circulation.

[8] Chartash EK, Lans DM, Paget SA (1989). Aortic insufficiency and mitral regurgitation in patients with systemic lupus erythematosus and the antiphospholipid syndrome. Am J Med.

[9] Cascella M, Rajnik M, Cuomo A (2025). Features, evaluation, and treatment of Coronavirus (COVID-19). StatPearls [Internet].

[10] Radic M, Martinovic Kaliterna D, Radic J (2013). Vascular manifestations of systemic lupus erythematosis. Neth J Med.

[11] Nikolopoulos D, Fanouriakis A, Boumpas DT (2019). Cerebrovascular events in systemic lupus erythematosus: diagnosis and management. Mediterr J Rheumatol.

